# Neural attention with character embeddings for hay fever detection from twitter

**DOI:** 10.1007/s13755-019-0084-2

**Published:** 2019-10-12

**Authors:** Jiahua Du, Sandra Michalska, Sudha Subramani, Hua Wang, Yanchun Zhang

**Affiliations:** Institute of Sustainable Industries & Liveable Cities, Victoria University, Melbourne, VIC Australia

**Keywords:** Pollen allergy, Hay fever, Twitter, Deep learning

## Abstract

The paper aims to leverage the highly unstructured user-generated content in the context of pollen allergy surveillance using neural networks with character embeddings and the attention mechanism. Currently, there is no accurate representation of hay fever prevalence, particularly in real-time scenarios. Social media serves as an alternative to extract knowledge about the condition, which is valuable for allergy sufferers, general practitioners, and policy makers. Despite tremendous potential offered, conventional natural language processing methods prove limited when exposed to the challenging nature of user-generated content. As a result, the detection of *actual* hay fever instances among the number of false positives, as well as the correct identification of non-technical expressions as pollen allergy symptoms poses a major problem. We propose a deep architecture enhanced with character embeddings and neural attention to improve the performance of hay fever-related content classification from Twitter data. Improvement in prediction is achieved due to the character-level semantics introduced, which effectively addresses the out-of-vocabulary problem in our dataset where the rate is approximately 9%. Overall, the study is a step forward towards improved real-time pollen allergy surveillance from social media with state-of-art technology.

## Introduction

Nearly one in five Australians suffered from hay fever in between 2014 and 2015 [1]. According to the World Health Organization [35], pollen allergy will only increase in prevalence and severity over the next decade, which leads to a global concern. Unsurprisingly, the accurate estimates of hay fever remain the top priority for Australian Institute of Health and Welfare. The traditional data sources for the condition scale evaluation include official statistics, general practitioner records, hospital admissions [34], antihistamine sales, etc. Still, the substantial time lag in the results reporting as well as the insufficient data granularity do not allow to obtain the accurate representation of pollen allergy prevalence and severity in real-time. Recently, social media data mining for public health surveillance has been growing in popularity in the research communities to account for the limitations of the existing methods. The critical challenge though lies in extracting the relevant information from highly unstructured data.

Social media platforms in particular abound in misspellings, abbreviations, and informal expressions. Neural network approaches have been applied lately in place of conventional machine learning techniques [17, 22, 25, 36] to account for the syntactic and semantic relationships between the words. Attention mechanism further enables to discriminate the words/phrases that contribute mostly towards the respective class assignment. To illustrate the problem, below presents the examples of relevant versus non-relevant posts, despite similar wording causing confusion in classification.

1. Hay fever-**related** content (Symptoms)
  My **eyes have been watering** and **I’ve been sneezing** heaps today... anyone else in Melbourne noticing their hay fever kicking in for the first time this spring?
2. Hay fever-**non-related** content (Advertisement)
  New one-off treatment for hay fever sufferers. It reduces symptoms by freezing the nerves that make you **sneeze**.

Traditional rule-based classifiers would consider the post containing term ‘*sneeze*’ relevant to the case study, thus overestimating the actual hay fever prevalence. On the other hand, numerous posts include pollen allergy-related terms expressed in the non-medical jargon, which proves impossible to detect using the pre-defined rules. Furthermore, the numerous Out-Of-Vocabulary (OOV) words prevalent on social media platforms significantly reduce the classification performance due to their absence in training dataset.

The primary objective of our study is to implement and validate the most recent neural networks approach with attention mechanism and character embeddings to the challenging problem of hay fever detection from Twitter. The posts are automatically classified into the 4 pre-defined classes (see Sect. 3.2) based on their relevance. Neural attention allows to increase the weight of the constituent parts of the posts that play a major role in final class prediction. The character embeddings on the other hand effectively tackle the OOV problem by utilizing the similar words syntax. We demonstrate an improvement in accuracy and macro-F1 of our proposed model as well as illustrate that our approach can both (i) capture the hay fever-related information in highly informal tweets (e.g. Symptoms, Treatments), and (ii) identify the non-relevant content (e.g. Advertisements, Warnings). The study provides the proof-of-concept of state-of-the-art techniques application in the context of pollen allergy towards improved public health surveillance from social media.

## Related work

### Health surveillance from social media

Individuals often prefer to share health-related experiences with peers, rather than during clinical studies, or even physicians [10]. In addition, the knowledge based solely on health practitioners’ reports and patients’ surveys tend to be generic and often limited in scope [8]. Furthermore, Cvetkovski et al. [9] reported that hay fever tend to be self-managed and availability of over-the-counter medications leads to bypassing the health care professionals, putting additional pressure on complementary data sources about the condition surveillance.

Given the limitations, social media has opened an enormous opportunity for public health surveillance from directly affected users. In particular, Twitter has recorded approximately three million active accounts since January 2018 in Australia [7]. Due to its short format, Twitter also encourages the high frequency of updates [18]. This in turn generates an abundance of data, commonly concerning the health-related matters [5]. As a result, Twitter has drawn attention from public health communities to answer numerous health-related questions [2].

In the case of pollen allergy surveillance, De Quincey et al. [11, 12] demonstrated that Twitter enables researchers to access information regarding the specific pollen allergy symptoms, as well as the medications usage and effectiveness. The comparison with UK Pollen Hotzones further proved that geolocated Twitter data is a good proxy for the condition prevalence estimation due to the similar distribution [12]. In the other study, Gesualdo et al. [14] observed the high correlation between pollen counts and tweets reporting hay fever incidents in the study conducted in the US. The results obtained serve as a proof of concept of the potential role of social media in signalling allergic symptoms and drug consumption trends.

### Hay fever prevalence in Australia

**Fig. 1:**
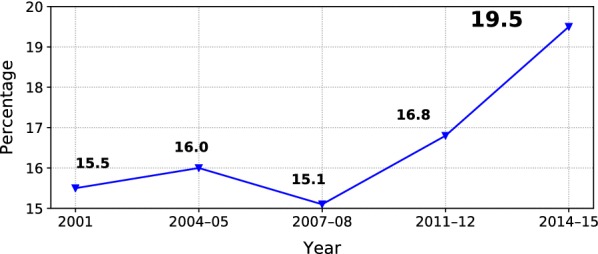
Prevalence of allergic rhinitis sufferers in Australia [1]

Three million Australian adults struggle through spring and summer with symptoms such as watery eyes, running nose, itchy throat, sneezing or irritability. Pollen allergy is also considered the most common chronic respiratory disease in Australia [1], posing a significant health and economic burden [9]. The quality of life of allergy sufferers is substantially reduced, affecting physical, psychological, and social functioning [35]. According to the National Health Survey conducted by the Australian Bureau of Statistics, which is shown in Fig. 1, the prevalence of allergic rhinitis among Australians has been measured over the past 15 years and indicated growth over time.

The exact estimations of hay fever prevalence proves a challenging task due to the limited resources, i.e. time- and cost-consuming official statistics, marketing surveys, pharmaceuticals data, etc. The usual peak in hay fever occurrences is observed around spring and summer period. However, climate changes observed are lengthening the pollen seasons as well as introducing an increased intensity of allergens, and unexpected new pollens in certain areas [35]. Additionally, the increasing air pollution, especially around urban areas further affects the respiratory health of the population. This in turn adds an uncertainty to the accurate hay fever prevalence estimation. The real-time monitoring proves invaluable for allergy sufferers, health practitioners, and policy makers.

### Deep learning in text classification

Previous studies on allergies surveillance from social media conducted in UK and US utilized either traditional machine learning classifiers, including Naive Bayes [6, 24] and lexicon-based approaches [14, 11, 12]. Despite the wealth of knowledge that social media offers, the natural language used still constitutes a major challenge in tweets analysis, and forms an obstacle in relevant information extraction [29]. For instance, the highly informal and continuously evolving vocabulary such as ‘*dribbling nose*’ and ‘*hay fever sob*’ prove difficult to classify as the potential symptoms and map to their medical equivalents, i.e. ‘*runny nose*’, ‘*watery eyes*’. Lack of advanced Natural Language Processing (NLP) techniques addressing the above mentioned issues leads to the limited applicability of the approaches in the case of the emerging symptoms/treatments, not identified a priori. Despite the existing shortcomings, no previous study applied deep learning to user-generated content classification in the context of hay fever. Furthermore, the performance improvement using neural attention is yet to be discovered in literature.

Deep learning has already proven successful in text classification tasks, outperforming the conventional machine learning techniques [16, 21, 26, 27], effectively capturing both syntactic (e.g. *allergy*, *allergic*, *allergen*, etc.) and semantic (e.g. *hay fever*, *pollen allergy*, *allergic rhinitis*) word dependencies. Also, deep learning alleviates the need for laborious and time-consuming manual feature engineering. The most distinctive features are extracted automatically from the raw input during the model training. The successful application of deep learning has been reported in numerous NLP tasks, including topic categorization [19], machine translation [31], sentence modelling [20], and Part-Of-Speech tagging [4].

Among many neural architectures, Recurrent Neural Networks (RNNs), in particular Long Short-Term Memory networks (LSTMs) [15] are widely implemented to model text sequences due to their capability in modeling long-range dependencies and historical information storage over time [32]. Attention mechanism can further boost the performance of RNNs by focusing on the time-steps that are most critical to the task [15]. In regular RNNs (without attention), the prediction is made using RNNs at the final time-step. With attention, RNNs save the output at every time-step, and the mechanism then selects and combines the most important outputs based on their relevance to the task [13]. The improved performance of RNNs with attention versus RNNs without attention was obtained in the case study of information extraction from cancer pathology reports [13].

## Methodology

### Data extraction

The data was sourced from the micro-blogging platform Twitter commonly used for public health surveillance purposes due to the high frequency of updates, convenience of geo-location, and the wide API availability. The geo-coordinates were set to the latitude and longitude of Alice Springs (centre of Australia), and the radius of 2000 miles. The tweets were extracted weekly using the R programming language and TwitteR package. The search criteria were ‘hayfever’ and ‘hay fever’ in order to ensure the high precision over recall of the returning tweets. The collection period was from June 1, 2018 to December 31, 2018, and covered the high pollen season in Australia. The data was obtained for 191 out of 214 days in total (89%). The tweets from the remaining 23 days were not captured due to technical issues. The mention of Twitter users are removed to ensure compliance to privacy and ethical considerations.

### Data annotation

The entire dataset containing 4148 posts was annotated by two researchers from health informatics. The annotation process followed the schema presented in Table 1, with tweet examples representative of each class. The respective categories were based on the relevancy to the aim of the study (1-most relevant, 4-least relevant). The two over-arching groups were distinguished, i.e. Informative and Non-Informative. The Cohen’s kappa statistic [3] was calculated to measure the inter-rater reliability. The score of $\kappa =0.82$ was obtained, which is considered significant based on the study by Viera et al. [33]. Any potential disagreements were either resolved by consensus, otherwise the ‘Unrelated/Ambiguous’ class was assigned.

**Table 1:** Annotation schema with the examples of tweets

Class	Description	Example
Informative		
1	Detailed personal reporting (symptoms, treatments, etc.)	My eyes have been watering and I’ve been sneezing heaps today...anyone else in Melbourne noticing their hay fever kicking in for the first time this spring?
2	Generic personal reporting	I wanted a Sunday morning lie-in, but hayfever is telling me different
Non-informative		
3	Warnings/news/marketing	Struggling with athsma or hayfever? Find out how a #saltlamp can help
4	Ambiguous/un-related	If I had hayfever I would simply buy some hay

### Data pre-processing

First, retweets and duplicate tweets are removed to prevent the dataset from redundant information. The remaining tweets are then tokenized into word sequences via a twitter-aware tokenizer. For each tokenized tweet: (i) all words are lowercased with the constituent URLs, punctuation, and stopwords removed; (ii) for each word, any characters repeating over three times in a word will be normalized by removing the remaining repetitions. As a result, the pre-processed dataset consists of 3862 tweets, covering 719 samples for Class 1, 1823 samples for Class 2, 938 samples for Class 3, and 382 samples for Class 4.

### Models for real-time hay fever detection

The real-time hay fever detection on tweets is regarded as a multi-class classification task. Given a set of tweets *D*, each tweet $d \in D$ is associated with one of the four predefined classes $y = \{1, 2, 3, 4\}$. The goal of the task is to learn automatically representative features that are able to correctly predict the corresponding class of a tweet. The architecture is presented as follows.

The architecture starts by encoding word-level semantic information of tweets. Let a tweet $d \in D$ be a sequence of *n* words $d = \{x_1, x_2, \ldots , x_n\}$. Each word $x \in V$ is associated with a *d*-dimensional embedding ${\mathbf {e}}_x$ to encode word-level semantics, which is stored in a word lookup table ${\mathbf {E}} \in {\mathbb {R}}^{|V| \times d}$, where *V* is the vocabulary of *D*.

In addition, character-level semantics is utilized to alleviate the OOV problem. Following [37], constituent characters of a word $x \in V$ are embedded into vectors, which are used to learn a *l*-dimensional character embedding ${\mathbf {e}}^{'}_{x}$ encoding sub-word information of the word via convolutional neural networks [23]. Each word *x* thus can be represented by concatenating both the corresponding word embedding ${\mathbf {e}}_{x}$ and character embedding ${\mathbf {e}}^{'}_{x}$. The representation of a tweet *d* is obtained by stacking its words:

1 $$\begin{aligned} {\mathbf {X}} = [ {\mathbf {e}} _{x_1} \oplus {\mathbf {e}} ^{'}_{x_2}, {\mathbf {e}} _{x_1} \oplus {\mathbf {e}} ^{'}_{x_2}, \ldots , {\mathbf {e}}_{x_n} \oplus {\mathbf {e}} ^{'}_{x_n} ], \end{aligned}$$

where $\oplus$ is the concatenation operator, the matrix ${\mathbf {X}} \in {\mathbb {R}}^{n \times (d+l)}$ represents a tweet, serving as one input sample for model fitting.

Given an input sample ${\mathbf {X}}$, a bidirectional LSTM is employed to obtain word annotations by summarizing contextual information of each word in a tweet.

2 $$\begin{aligned}{}[ \overrightarrow{\mathbf{h }}_1, \overrightarrow{\mathbf{h }}_2, \ldots , \overrightarrow{\mathbf{h }}_n ]&= \overrightarrow{\text {LSTM}}({\mathbf {X}} ), \end{aligned}$$

3 $$\begin{aligned}{}[ \overleftarrow{\mathbf{h }}_1, \overleftarrow{\mathbf{h }}_2, \ldots , \overleftarrow{\mathbf{h }}_n ]&= \overleftarrow{\text {LSTM}}({\mathbf {X}} ). \end{aligned}$$

The final annotation of a word ${\mathbf {h}} = \overrightarrow{\mathbf{h }} \oplus \overleftarrow{\mathbf{h }}$ results from concatenating the forward hidden state $\overrightarrow{\mathbf{h }} \in {\mathbb {R}}^{m}$ and backward hidden state $\overleftarrow{\mathbf{h }} \in {\mathbb {R}}^{m}$. The sentence embedding ${\mathbf {s}}$ representing the tweet is then constructed using the annotations via attention mechanisms:

4 $$\begin{aligned} {\mathbf {z}} _i&= \text {tanh}({\mathbf {W}}_a^{\top }{} {\mathbf {h}}_i + {\mathbf {b}}_a), \end{aligned}$$

5 $$\begin{aligned} \alpha _i&= \frac{\exp ({\mathbf {z}}_i^{\top }\hat{\mathbf{h }})}{\sum ^n_{j=1}\exp ({\mathbf {z}}_j^{\top }\hat{\mathbf{h }})}, \end{aligned}$$

6 $$\begin{aligned} {\mathbf {s}}&= \sum ^n_{i=1} \alpha _i {\mathbf {h}}_i, \end{aligned}$$

where ${\mathbf {W}}_a$, ${\mathbf {b}}_a$, and $\hat{\mathbf{h }}$ are learned parameters. The sentence embedding ${\mathbf {s}} \in {\mathbb {R}}^{m}$ is forwarded into an output layer to predict the probability distribution ${\mathbf {p}}$ over the predefined classes.

7 $$\begin{aligned} {\mathbf {p}} = \text {softmax}({\mathbf {W}}_o^{\top }{} {\mathbf {s}} + b_o). \end{aligned}$$

The model is trained to minimize the cross entropy between true labels and the predicted labels across tweets in the dataset *D*.

8 $$\begin{aligned} \mathcal {L} = -\sum _{d \in D}\log {\mathbf {p}}_d(y_d), \end{aligned}$$

where $y_d$ is the true label and ${\mathbf {p}}_d(y_d)$ is the probability of the true label. For simplicity, the attention-based bidirectional LSTM model is henceforth called BILSTM + ATT, whereas our architecture BILSTM + ATT + CHAR.

### Model training and evaluation

The neural models are evaluated using stratified 10-fold cross validation. As Table 2 shows, the dataset has on average approximately 9% missing words across testing folds, whereas the rate of missing characters is far lower.

**Table 2:** OOV rate of words and characters across testing folds

Fold	1	2	3	4	5	6	7	8	9	10	Average
OOV words	8.64	8.52	9.26	8.19	9.42	9.08	9.79	7.57	8.88	9.89	8.92
OOV characters	0.07	0.07	0.03	0.06	0.03	0.05	0.01	0.04	0.03	0.06	0.04

The following hyper-parameters are used. The dimensionality of word and character embeddings $d=l=50$. The word lookup table ${\mathbf {E}}$ is initialized with the GloVe embeddings pre-trained [28] on two billion tweets. The number and region size of kernels used to extract character embeddings are set to 100 and 5, respectively. The number of LSTM units is set to $m=50$. For each fold, 10% of the training data is randomly selected for validation, with mini-batch size of 64 and the Adam optimizer updating neural weights. Early stopping is employed to prevent over-fitting when the validation loss stops improving for 10 epochs. Each model is trained 5 times to report average results for model robustness under random parameter initialization.

## Result analysis

### Quantitative evaluation

Both accuracy and macro-F1 are used to evaluate model performance on the dataset with imbalanced labels. Table 3 demonstrates that incorporating character embeddings that encode sub-word information outperforms the original attention-based model by about 2% in accuracy. The improvement in macro-F1 is even more with almost 3%, suggesting that sub-word information is useful in hay fever prediction for classes with fewer training samples.

**Table 3:** The performance of model variants

Model	Accuracy	Macro-F1
BILSTM + ATT	77.72	72.80
BILSTM + ATT + CHAR	79.51	75.67

### Qualitative analysis

#### Attention mechanism

has the advantage of generating higher weights to the words that contribute mostly towards the relevant class assignment. Table 4 produces examples of posts with the attention maps overlaid on the text. Higher color intensity indicates greater attention score, and thus more impact on the final class prediction. For instance, in the posts from Class 1 (Symptoms/Treatments), the higher weight was assigned to the words ‘crying’ (= watery eyes), and ‘meds’ (= medications), despite no obvious symptom/treatment indication. In the case of Class 2 (Generic reporting), hay fever-related terms (e.g. ‘season’, ‘pollen’) were attended more in model learning. Also, the term ’windy’ obtained a higher score, which can be considered intuitive as pollen spreads more intensively during strong winds. As for Class 3 (Warnings/News/Marketing), the more formal and non allergy-related words such as ‘surge’ and ‘news’ mostly contributed to the News/Warnings prediction. Finally, in the posts from Class 4 (Ambiguous/Un-related) the irrelevant terms from hay fever detection point of view were highlighted, i.e. the words ‘parliament’ or ‘bottle’ were un-seen in the hay fever-related content, thus became decisive of Ambiguous/Unrelated class assignment.

**Table 4:**
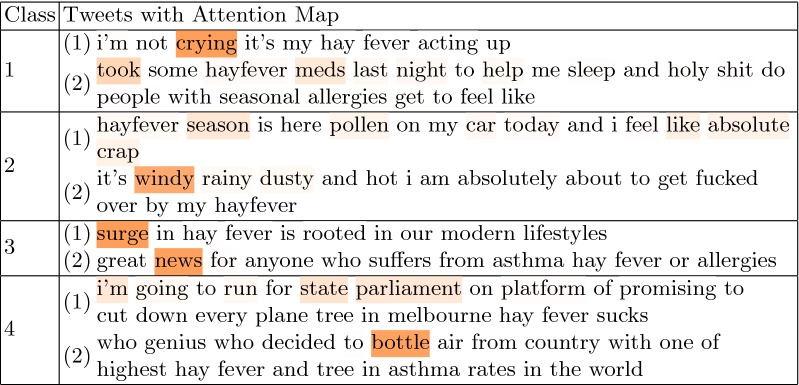
The examples of post with the attention maps

Color intensity indicates the weight attributed to each word towards the respective class assignment

#### Character embeddings

encode character-level features of the words that share similar prefix/suffix [30], and obtain closer representation among words within the same class based on their characters [37]. The implementation of character embeddings is mainly driven by the occurrence of OOV problem, which is particularly visible in the case of social media data due to the number of grammatical errors (e.g. ‘athsma’), abbreviated forms (e.g. ‘meds’), jargonic expressions (e.g. ‘sniffling’), etc.

In Table 5, the examples of posts with OOV have been illustrated, including the respective class probabilities obtained for both models. The probabilities for the model with character embeddings were higher, demonstrating greater confidence towards the actual class prediction. This can be attributed to the OOV recognition and mapping, where similar syntactical structure was utilized, i.e. **med** = **med**ication, **ads** = **ad**vertisement**s**, **probs** = **prob**ably, etc. Such an approach proves particularly beneficial in the context of health surveillance, where complex medical terminology (e.g. Allergic Rhinitis), treatment names (e.g. antihistamines), and medication brands (e.g. Loratadine) are highly prone to frequent mispronunciation on social media platforms.

**Table 5:** The examples of posts with OOV words and their respective predictions probabilities for BILSTM + ATT (A) and BILSTM + ATT + CHAR (B)

Class	Prob. (A)	Prob. (B)	Post
1	0.60	0.99	**um** i’m still sick also hay fever kicking **n** eyes **r** burning **n** red day **v** self
1	0.97	0.98	**aaahhh** hayfever season allow play song people ...sneezes minutes straight
1	0.20	0.99	seriously one bizarre moments life told buy usual effective hayfever **med**
2	0.03	0.50	**probs** hay fever
2	0.94	1.00	canny sun **coz** hay fever
2	0.80	1.00	im thinking immune **aka** hayfever due pollen air last couple days
4	0.26	0.67	last one like **ads** hayfever sufferers going mars avoid allergens

## Conclusions

Pollen allergy is a major health and economic burden, impacting the lives of approximately 20% of Australian population, in particular working-aged adults. The real-time monitoring of the condition prevalence and severity is currently unavailable. Twitter data has been proven to be a valuable source of information on emerging symptoms as well as treatments usage from directly affected individuals. The challenging nature of user-generated content (i.e. misspellings, abbreviations, jargon) poses significant limitation in actionable knowledge extraction. Deep learning is currently the state-of-the-art in NLP tasks. We employ the neural networks model with the attention mechanism to account for the fact that each word contributes differently towards final class assignment. We further improve the classification accuracy of the model with character embeddings implementation, which effectively addresses the OOV problem. We also discuss the inner workings of the attention mechanism as well as character embeddings on the examples of posts to facilitate finding interpretability. Taken together, the study demonstrates and validates the practical application of state-of-art deep learning in the context of pollen allergy surveillance from social media.
